# Diagnostic and therapeutic characteristics of diabetes mellitus and risk of out-of-hospital cardiac arrest

**DOI:** 10.1038/s41598-022-05390-w

**Published:** 2022-01-25

**Authors:** Jeong Ho Park, Young Sun Ro, Sang Do Shin, Kyoung-Chul Cha, Kyoung Jun Song, Sung Oh Hwang, For the phase II Cardiac Arrest Pursuit Trial with Unique Registry and Epidemiologic Surveillance (CAPTURES-II) investigators, Sung Oh Hwang, Sang Do Shin, Mi Jin Lee, Jong-Hak Park, Su Jin Kim, Sung Bum Oh, Jonghwan Shin, Seung Min Park, Min Seob Sim, Won Young Kim, In-Cheol Park, Hyun Ho Ryu, Yeonho You, Sang-Chul Kim, Ju Ok Park

**Affiliations:** 1 Department of Emergency Medicine, Seoul National University College of Medicine and Seoul National University Hospital, Seoul, Korea; 2 Laboratory of Emergency Medical Services, Seoul National University Hospital Biomedical Research Institute, 101 Daehak-Ro, Jongno-Gu, Seoul, 03080 Korea; 3 Department of Emergency Medicine, Yonsei University Wonju College of Medicine, Wonju, Korea; 4 Department of Emergency Medicine, Seoul National University Boramae Medical Center, Seoul, Korea; 5 Yonsei University Wonju Severance Christian Hospital, Wonju, Korea; 6 Seoul National University Hospital, Seoul, Korea; 7 Kyungpook National University Hosptial, Daegu, Korea; 8 Korea University Ansan Hospital, Ansan, Korea; 9 Korea University Anam Hospital, Seoul, Korea; 10 Dankook University Hospital, Yongin, Korea; 11 Seoul National University Boramae Medical Center, Seoul, Korea; 12 Seoul National University Bundang Hospital, Seongnam, Korea; 13 Sungkyunkwan University Samsung Medical Center, Seoul, Korea; 14 Ulsan University Asan Medical Center, Seoul, Korea; 15 Yonsei University Severance Hospital, Seoul, Korea; 16 Chonnam National University Hospital, Gwangju, Korea; 17 Chungnam National University Hospital, Daejeon, Korea; 18 Chungbuk National University Hospital, Cheongju, Korea; 19 Hallym University Dongtan Sacred Heart Hospital, Chuncheon, Korea

**Keywords:** Cardiovascular diseases, Endocrine system and metabolic diseases

## Abstract

This study aimed to evaluate the risks of diabetes mellitus (DM) on out-of-hospital cardiac arrest (OHCA) and to investigate whether the risks of DM on OHCA varied according to the diagnostic and therapeutic characteristics of diabetes. We conducted a multicenter prospective case–control study in 17 University hospitals in Korea from September 2017 to December 2020. Cases were EMS-treated OHCA patients aged 20 to 79 with a presumed cardiac etiology. Community-based controls were recruited at a 1:2 ratio after matching for age, sex, and urbanization level of residence. A structured questionnaire and laboratory findings were collected from cases and controls. Multivariable conditional logistic regression analyses were conducted to estimate the risk of DM on OHCA by characteristics. A total of 772 OHCA cases and 1544 community-based controls were analyzed. A total of 242 (31.3%) OHCAs and 292 (18.9%) controls were previously diagnosed with DM. The proportions of type I DM (10.7% vs. 2.1%) and insulin therapy (15.3% vs. 6.5%) were higher in OHCAs with DM than in controls with DM. The duration of DM was longer in OHCAs than in controls (median 12 vs. 7 years). DM was associated with an increased risk of OHCA (aOR (95% CI), 2.13 (1.64–2.75)). Compared to the no diabetes group, the risks of OHCA increased in the diabetes patients with type I DM (5.26 (1.72–16.08)) and type II DM group (1.63 (1.18–2.27)), a long duration of DM prevalence (1.04 (1.02–1.06) per 1-year prevalence duration), and a high HbA1c level (1.38 (1.19–1.60) per 1% increase). By treatment modality, the aOR (95% CI) was lowest in the oral hypoglycemic agent (1.47 (1.08–2.01)) and highest in the insulin (6.63 (3.04–14.44)) groups. DM was associated with an increased risk of OHCA, and the risk magnitudes varied according to the diagnostic and therapeutic characteristics.

## Introduction

Diabetes mellitus (DM) poses significant health burden worldwide, and the burdens of disease are increasing globally^1,2^. In 2017, over 6% of the world’s population was affected by DM, and over 1 million deaths per year could be attributed to diabetes alone^1^. The strong association between DM and cardiovascular disease is well known^3^, and cardiovascular disease is the most critical cause of mortality and morbidity in patients with diabetes^4^. Both microvascular and macrovascular complications of diabetes explain the close link between DM and fatal cardiovascular disease^3^.

Previous studies reported that DM increased the risk of sudden cardiac arrest, including out-of-hospital cardiac arrest (OHCA)^5–8^. Several mechanisms have been proposed for these associations, including arrhythmogenic potentials associated with autonomic neuropathy/prolonged QT interval/reduced heart rate variability, atherogenic events associated with myocardial ischemia or hypercoagulable state, and direct cardiac pathology, including diabetic cardiomyopathy^6,8–11^.

OHCA poses a significant health burden worldwide due to its high mortality and poor prognosis^12–14^. The prevalence of DM in OHCA patients is higher than that in the general population, and the survival outcomes of OHCAs with diabetes are poor^15,16^. A better understanding of the risks of DM on cardiac arrest according to the characteristics of diabetes would be essential to screening high-risk populations among patients with DM and to developing effective preventive strategies for reducing the health burden of fatal cardiovascular complications, such as OHCA.

Diabetes is a chronic disease, and the diagnostic and therapeutic characteristics are not homogenous among patients with the condition. However, the risk variations of DM on OHCA by its characteristics are not well explained, whereas the relationship between DM itself and OHCA is well established. Understanding the risk variations of OHCA for patients with diabetes could contribute to developing prevention strategies for sudden cardiac arrests caused by diabetes. The aims of this study were to evaluate the association between diagnosed DM and OHCA and to investigate whether the risk magnitudes of OHCA were different according to the diagnostic and therapeutic characteristics of diabetes, including the type of DM, duration of prevalence, treatment modality, and HbA1c level.

## Methods

### Study design, setting, and data source

This study was a multicenter case–control study using the phase II Cardiac Arrest Pursuit Trial with Unique Registration and Epidemiologic Surveillance (CAPTURES-II) project in Korea.

The CAPTURES project was a prospective multicenter project aimed at identifying the risk factors for OHCA incidence and evaluating determinants of good prognosis with long-term follow-up funded by the Korea Disease Prevention and Control Agency. Phase I of the CAPTURES project was a prospective hospital-based OHCA registration study conducted at 27 emergency departments (EDs) from January to December 2014. A detailed description of phase I of the CAPTURE study was previously described^5^.

The CAPTURES-II study was a prospective case–control study conducted at the EDs of 17 university hospitals from September 2017 to December 2020. The project was subjected to OHCA patients transported to the study EDs by emergency medical service (EMS) with resuscitation efforts (EMS-treated OHCA) and patients who had a presumed cardiac etiology as identified by emergency physicians in each ED. The CAPTURES-II project included face-to-face interviews (for patient sociodemographics, health behaviors, stress, and comorbidities), medical record reviews (for Utstein templates, laboratory tests, cardiac examinations, and short- and long-term outcomes), and blood sample registration for biomarker evaluations for OHCA patients. For community-based controls, face-to-face interviews for the same information of OHCA patients and blood sample registration for laboratory tests and biomarker evaluations were conducted. All data collected from participating hospitals were transferred to the Data Quality Management Committee (DQMC), where quality-control checks and statistical analysis were performed. DQMC gave feedback to each center coordinator on the quality management of the CAPTURES data through monthly quality control meetings. This study protocol is registered at ClinicalTrials.gov (NCT03700203).

### Cases

For the CAPTURES-II project, EMS-treated OHCA patients aged 20–79 years who had a presumed cardiac etiology were prospectively recruited. The project excluded OHCA patients of noncardiac etiology, including trauma, drowning, poisoning, burn, asphyxia, or hanging, and OHCA cases with a terminal illness, under hospice care, with a ‘Do Not Resuscitate’ card, with pregnancy, and living alone or experiencing homelessness without reliable information sources. OHCA patients with unknown information on DM were excluded (n = 26).

### Controls

Community-based controls were recruited from 1 metropolitan university hospital and 1 nonmetropolitan university hospital. One center located in a metropolitan area (Seoul) collected metropolitan controls, and the other center located in a nonmetropolitan area (Wonju) collected nonmetropolitan controls. By collaborating with public health centers or various community centers, the CAPTURES-II project and control recruitment were promoted, and voluntary applicants were recruited as the control group. Age (5-year intervals), sex, and urbanization level of residence (metropolitan vs nonmetropolitan) matched controls were recruited at a 1:2 ratio per case.

### Variables and measurements

The main exposure was physician-diagnosed DM prior to study enrollment as measured via face-to-face interviews.

The CAPTURES-II project used the same structured questionnaires for case and controls. Emergency physicians at study EDs collected the information during a face-to-face interview with the patients’ family for the OHCA case group and study participants for the community-based control group. Information about demographics (age, sex, and residence area) and comorbidities (diabetes, hypertension, myocardial infarction, stroke, dyslipidemia, and arrhythmia) was collected. Only comorbidities that were diagnosed by doctors in hospitals or clinics were documented, and the exact question was determined in the protocol to standardize the survey process. For example, “Have you (or the OHCA patient) ever been diagnosed with an arrhythmia by a doctor?” was used to identify arrhythmia, and if yes, they were classified into the arrhythmia group regardless of specific type of arrhythmia. Health behaviors, including current smoking status, obesity, and regular exercise during the last year, were also noted. Current smoking status was defined as smoking more than 1 cigarette per day within the past month. Regular exercise during the last year was defined as moderate- and vigorous-intensity exercise at least once per week in the last year.

For participants with diabetes, information on the type of DM (Type I or Type II), duration of diabetes prevalence, and DM treatment modality (insulin, oral hypoglycemic agent, lifestyle modification, and no treatment) were collected. Patients receiving combined therapy with insulin and oral hypoglycemic agents were classified as the insulin group. The duration of diabetes was categorized into 5 groups: 0–1 years, 2–3 years, 4–10 years, 11–18 years, and 19– years.

For cases, blood samples during initial management were collected, and hemoglobin A1c (HbA1c) measurements were conducted at each hospital’s laboratory department. For controls, laboratory tests, including HbA1c, were performed with face-to-face interviews at the time of enrollment. HbA1c levels were categorized into three groups: 0–5.6%, 5.7–6.4% and 6.5–%.

### Statistical analysis

Demographic findings of OHCA cases and community-based controls were described. Continuous variables were compared using the Mann–Whitney U test, and categorical variables were compared using the chi-square test or Fisher’s exact test. Multiple imputations were conducted for 9 risk factors, including hypertension (n = 13, 7 cases and 6 controls), myocardial infarction (n = 28, 9 and 19), stroke (n = 28, 11 and 17), dyslipidemia (n = 49, 31 and 18), arrhythmia (n = 36, 22 and 14), current smoking (n = 20, 19 and 1), obesity (n = 41, 24 and 17), and regular exercise during the last year (n = 45, 41 and 4).

We used conditional logistic regression for the matched case–control dataset. Adjusted odds ratios (aORs) and 95% confidence intervals (CIs) were calculated to estimate the effect of DM on OHCA risk. All variables included in the final model were assessed for multicollinearity. No significant collinearity was detected.

Analyses for risk variations of DM on OHCA according to diagnostic characteristics (type of DM and duration of diabetes prevalence) and therapeutic characteristics (treatment modality and HbA1c level) of diabetes were conducted. For each characteristic of diabetes, 1:2 matched case–control sets were extracted and analyzed where information on the characteristics of DM was not missing for both 1 case and 2 matched controls: type of DM (662 cases and 1324 controls), duration of diabetes (655 patients and 1310 controls), treatment modality (724 cases and 1448 controls), and HbA1c level (382 cases and 764 controls).

Sensitivity analyses without imputation were completed for the main analyses. Statistical analyses were performed using SAS 9.4. We considered a 2-sided *P* < 0.05 to be statistically significant.

### Ethic statements

The study was approved by the ethics committees of all 17 participating university hospitals. All participants, or their proxy, provided written informed consent before taking part in the study. All methods were performed in accordance with relevant guidelines and regulations.

### Ethic statements

The study was approved by the ethics committees of all 17 participating university hospitals (IRB No: Chonnam National University Hospital, CNUH-2017-285; Chungbuk National University Hospital, CBNUH2017-09-009-001; Chungnam National University Hospital, CNUH2017-10-027; Dankook University Hospital, DKUH2018-12-019; Hallym University Kangnam Sacred Heart Hospital, HKS2018-02-016; Hallym University Dongtan Sacred Heart Hospital, HDT2017-10-002; Korea University Anam Hospital, 2018AN0148; Korea University Ansan Hospital, AS17174; Kyungpook National University Hosptial, KNUH2017-10-035-006; Seoul National University Boramae Medical Center, 20171123/30-2017-66/123; Seoul National University Bundang Hospital, B-1711/430-304; Seoul National University Hospital, H-1709-053-883; Soonchunhyang University Bucheon Hospital, SCHBC2018-02-014-002; Sungkyunkwan University Samsung Medical Center, SMC2018-08-121; Ulsan University Asan Medical Center, S2018-1805-0001; Yonsei University Severance Hospital, 4-2017-1201; Yonsei University Wonju Severance Christian Hospital, CR317101). All participants, or their proxy, provided written informed consent before taking part in the study. This study is registered at ClinicalTrials.gov (NCT03700203).

## Results

### Demographic findings

A total of 772 EMS-treated OHCA cases and 1544 community-based matched controls were analyzed in the primary analysis. The characteristics of the OHCA case and matched control groups are shown in Table 1. Of 772 OHCA cases and 1544 matched controls, 242 (31.3%) cases and 292 (18.9%) controls were diagnosed with DM (*P* < 0.01). For comorbidities, the prevalences of hypertension, myocardial infarction, stroke, and arrhythmia were significantly higher in OHCA cases than in controls, whereas the prevalence of dyslipidemia was higher in controls than in OHCA cases (all *P* < 0.01). Among OHCA cases and matched controls diagnosed with DM, the proportion of type I DM was significantly higher in OHCA cases than in controls (10.7% vs. 2.1%, *P* < 0.01), and the median (IQR) duration of diabetes was longer in OHCA cases than in controls (12 (7–19 )vs. 7 (3–15 )years, *P* < 0.01). The proportion of insulin therapy was higher in OHCA cases than in controls with DM (15.3% vs. 6.5%, *P* < 0.01). The medians (IQRs) of HbA1c levels were 7.0% (6.1–8.2) for OHCA cases with DM and 6.7% (6.3–7.4) for controls with DM (*P* = 0.16). The characteristics of study population by type of DM are shown in the Supplementary Table 1.

**Table 1:** Characteristics of the out-of-hospital cardiac arrest case group and age-, sex-, and urbanization level-matched control group.

	Total	OHCA case	Control	*P*
	N (%)	N (%)	N (%)	
Total	2316	772	1544	
Sex, male	1671 (72.2)	557 (72.2)	1114 (72.2)	1.00
Age, mean (SD), year	58.2 (12.0)	58.2 (12.0)	58.3 (12.1)	0.88
Age, year				1.00
19–29	45 (1.9)	15 (1.9)	30 (1.9)	
30–39	132 (5.7)	44 (5.7)	88 (5.7)	
40–49	372 (16.1)	124 (16.1)	248 (16.1)	
50–59	573 (24.7)	191 (24.7)	382 (24.7)	
60–69	777 (33.5)	259 (33.5)	518 (33.5)	
70–79	417 (18.0)	139 (18.0)	278 (18.0)	
Urbanization level of residence				1.00
Metropolitan	1152 (49.7)	384 (49.7)	768 (49.7)	
**Health behavior**				
Current smoker	588 (25.4)	293 (38.0)	295 (19.1)	< 0.01
Obesity	217 (9.4)	32 (4.1)	185 (12.0)	< 0.01
Regular exercise during the last year	1195 (51.6)	223 (28.9)	972 (63.0)	< 0.01
**Comorbidity**				
Diabetes mellitus	534 (23.1)	242 (31.3)	292 (18.9)	< 0.01
Hypertension	911 (39.3)	355 (46.0)	556 (36.0)	< 0.01
Myocardial infarction	80 (3.5)	64 (8.3)	16 (1.0)	< 0.01
Stroke	102 (4.4)	59 (7.6)	43 (2.8)	< 0.01
Dyslipidemia	504 (21.8)	112 (14.5)	392 (25.4)	< 0.01
Arrhythmia	105 (4.5)	60 (7.8)	45 (2.9)	< 0.01
Diabetes mellitus				
Diagnosis, subtotal	534	242	292	
Type of diabetes				< 0.01
Type I	32 (6.0)	26 (10.7)	6 (2.1)	
Type II	358 (67.0)	152 (62.8)	206 (70.5)	
Unknown	144 (27.0)	64 (26.4)	80 (27.4)	
Duration of diabetes, years				< 0.01
0–3	114 (21.3)	19 (7.9)	95 (32.5)	
4–10	130 (24.3)	49 (20.2)	81 (27.7)	
11–18	77 (14.4)	33 (13.6)	44 (15.1)	
19–	85 (15.9)	40 (16.5)	45 (15.4)	
Unknown	128 (24.0)	101 (41.7)	27 (9.2)	
Median (IQR)	10 ^3–17^	12 ^7–19^	7 ^3–15^	< 0.01
Treatment of diabetes				< 0.01
No treatment	28 (5.2)	11 (4.5)	17 (5.8)	
Lifestyle modification	30 (5.6)	12 (5.0)	18 (6.2)	
Oral hypoglycemic agent	372 (69.7)	145 (59.9)	227 (77.7)	
Insulin	56 (10.5)	37 (15.3)	19 (6.5)	
Unknown	48 (9.0)	37 (15.3)	11 (3.8)	
HbA1c, %				0.15
0–5.6	37 (6.9)	16 (12.9)	21 (7.2)	
5.7–6.4	109 (20.4)	29 (23.4)	80 (27.4)	
6.5–	270 (50.6)	79 (63.7)	191 (65.4)	
Unknown	118 (22.1)	118 (48.8)	0 (0.0)	
Median (IQR)	6.7 (6.2–7.6)	7.0 (6.1–8.2)	6.7 (6.3–7.4)	0.16

IQR, interquartile range.

Of the 772 OHCA cases, 528 (68.3%) cases were witnessed, 245 (31.7%) cases occurred in public places, 481 (62.3%) cases received bystander cardiopulmonary resuscitation, 334 (43.3%) cases showed initial shockable rhythm at scene, and 386 (50.0%) cases received prehospital defibrillation. Survival to discharge rates were 46.8% (361/772) for all OHCA cases, 38.8% (94/242) for OHCAs with DM, and 50.4% (267/530) for OHCAs without DM, respectively.

### Main analyses

Using multivariable conditional logistic regression models, diagnosed DM was associated with an increased risk for OHCA after adjustment for comorbidities and health behaviors (aOR (95% CI): 2.13 (1.64–2.75)) (Table 2).

**Table 2:** Risk of diabetes mellitus for the out-of-hospital cardiac arrest among the study population.

	OHCA case/control	Odds ratio (95% CI)		
		Model 1	Model 2	Model 3
Diabetes mellitus	772/1544			
No diabetes	530/1252	1.00	1.00	1.00
Diagnosed	242/292	2.25 (1.81–2.80)	2.15 (1.69–2.73)	2.13 (1.64–2.75)

CI, confidence interval.

Model 1: Unadjusted conditional logistic regression.

Model 2: Conditional logistic regression adjusted for comorbidities (hypertension, myocardial infarction, stroke, dyslipidemia, and arrhythmia).

Model 3: Conditional logistic regression adjusted for comorbidities (hypertension, myocardial infarction, stroke, dyslipidemia, and arrhythmia) and health behaviors (current smoking status, obesity, and regular exercise during the last year).

To examine the variations in the risk magnitude of DM on OHCA according to the diagnostic and therapeutic characteristics, aORs with 95% CIs were calculated for subgroups where information on the type of DM, duration of illness, type of treatment, and HbA1c level was not missing. In terms of the type of diabetes, the aOR (95% CI) was the highest in the type I DM group (5.26 (1.72–16.08)), followed by the type II DM group (1.63 (1.18–2.27)), compared to the no diabetes group. For the duration of diabetes, patient groups with diabetes prevalence longer than 10 years were associated with increased risks of OHCA compared to the no diabetes group (aORs (95% CIs): 2.25 (1.21–4.18) for 11–18 years group and 2.24 (1.23–4.09) for 19– years group, respectively).

In terms of the treatment modality of diabetes, the aOR (95% CI) was lowest in the oral hypoglycemic agent group (1.47 (1.08–2.01)) and highest in the insulin group (6.63 (3.04–14.44)) compared to the no diabetes group. Regarding HbA1c level, the aOR (95% CI) of a 1% increase in HbA1c level was 1.38 (1.19–1.60). There was a dose-dependent relationship between HbA1c level and OHCA: aORs (95% CIs) were 2.17 (1.45–3.24) for the group whose HbA1c level was over 6.5% and 1.60 (1.12–2.29) for the HbA1c 5.7–6.4% group compared to the HbA1c 0–5.6% group (Table 3).

**Table 3:** Risk of characteristics of diabetes mellitus for out-of-hospital cardiac arrest among the study population.

	OHCA Case/Control	Odds ratio (95% CI)^†^		
		Model 1	Model 2	Model 3
Total	772/1544			
Type of diabetes, subtotal	662/1324			
No diabetes	530/1169	1.00	1.00	1.00
Type I DM	17/6	6.71 (2.62–17.18)	6.52 (2.23–19.07)	5.26 (1.72–16.08)
Type II DM	115/149	1.86 (1.40–2.47)	1.71 (1.26–2.31)	1.63 (1.18–2.27)
Duration of diabetes, subtotal	655/1310			
No diabetes	530/1140	1.00	1.00	1.00
0–3 years	19/66	0.67 (0.39–1.13)	0.61 (0.34–1.07)	0.57 (0.31–1.05)
4–10 years	34/50	1.59 (1.01–2.52)	1.37 (0.83–2.27)	1.30 (0.75–2.24)
11–18 years	33/26	2.92 (1.71–4.97)	2.68 (1.50–4.79)	2.25 (1.21–4.18)
19– years	39/28	3.29 (1.97–5.49)	2.39 (1.37–4.18)	2.24 (1.23–4.09)
Duration of diabetes (per year) ‡	655/1310	1.06 (1.04–1.08)	1.05 (1.03–1.07)	1.04 (1.02–1.06)
Treatment of diabetes, subtotal	724/1448			
No diabetes	530/1200	1.00	1.00	1.00
No treatment with diabetes	11/9	3.05 (1.25–7.46)	2.80 (1.04–7.48)	2.76 (1.01–7.58)
Lifestyle modification	12/18	1.68 (0.80–3.54)	2.34 (1.07–5.12)	2.62 (1.14–6.06)
Oral hypoglycemic agents	139/207	1.72 (1.33–2.23)	1.54 (1.15–2.04)	1.47 (1.08–2.01)
Insulin	32/14	5.75 (3.02–10.94)	6.94 (3.45–13.96)	6.63 (3.04–14.44)
HbA1c, subtotal	382/764			
0–5.6%	179/455	1.00	1.00	1.00
5.7–6.4%	101/178	1.56 (1.14–2.13)	1.58 (1.14–2.20)	1.60 (1.12–2.29)
6.5– %	102/131	2.26 (1.61–3.17)	2.49 (1.73–3.58)	2.17 (1.45–3.24)
HbA1c (per one percent)	382/764	1.43 (1.25–1.63)	1.48 (1.29–1.70)	1.38 (1.19–1.60)

CI, confidence interval; DM, diabetes mellitus; HbA1c, hemoglobin A1c.

Model 1: Unadjusted conditional logistic regression.

Model 2: Conditional logistic regression adjusted for comorbidities (hypertension, myocardial infarction, stroke, dyslipidemia, and arrhythmia) .

Model 3: Conditional logistic regression adjusted for comorbidities (hypertension, myocardial infarction, stroke, dyslipidemia, and arrhythmia) and health behaviors (current smoking status, obesity, and regular exercise during the last year).

^†^Separate models were conducted according to each characteristic of DM (type of diabetes, duration of diabetes, treatment of diabetes, and hemoglobin A1c), and only one characteristic of DM was included in each model.

^‡^For patients without diabetes, the duration of diabetes (continuous variable) was zero.

In sensitivity analyses, similar results were observed for the study populations without imputation (Table 4).

**Table 4:** Sensitivity analyses for the study population without covariate imputation.

	OHCA case/Control	Odds ratio (95% CI)
Diabetes mellitus	627/1254	
No diabetes	442/1033	1.00
Diagnosed	185/221	2.08 (1.55–2.78)
Type of diabetes, subtotal	547/1094	
No diabetes	442/970	1.00
Type I DM	11/5	4.59 (1.45–14.53)
Type II DM	94/119	1.67 (1.18–2.36)
Duration of diabetes, subtotal	534/1068	
No diabetes	442/945	1.00
0–3 years	16/47	0.77 (0.39–1.53)
4–10 years	27/42	1.19 (0.65–2.20)
11–18 years	21/16	1.77 (0.84–3.73)
19– years	28/18	2.03 (0.97–4.25)
Duration of diabetes (per year)^†^	534/1068	1.04 (1.01–1.07)
Treatment of diabetes, subtotal	588/1176	
No diabetes	442/996	1.00
No treatment with diabetes	7/9	2.26 (0.73–6.97)
Lifestyle modification	10/14	2.43 (0.97–6.08)
Oral hypoglycemic agents	106/148	1.53 (1.07–2.18)
Insulin	23/9	6.13 (2.43–15.48)
HbA1c, subtotal	309/618	
0–5.6%	152/377	1.00
5.7–6.4%	79/147	1.44 (0.97–2.14)
6.5– %	78/94	2.06 (1.31–3.22)
HbA1c (per one percent)	309/618	1.34 (1.14–1.58)

CI, confidence interval; DM, diabetes mellitus; HbA1c, hemoglobin A1c.

Conditional logistic regression adjusted for comorbidities (hypertension, myocardial infarction, stroke, dyslipidemia, and arrhythmia) and health behaviors (current smoking status, obesity, and regular exercise during the last year).

^†^For patients without diabetes, the duration of diabetes was zero.

## Discussion

This study found that diagnosed DM increases the risk of OHCA, and the risk magnitudes varied according to the diagnostic and therapeutic characteristics of DM using the CAPTURES-II project, a multicenter prospective case–control study. Regarding the type of diabetes, the risk of OHCA in the type I DM group was greater than that in the type II DM group compared to the no diabetes group. By treatment modality, the risk was highest in the insulin group, followed by the no treatment and lifestyle modification groups, and lowest in the oral hypoglycemic agent group. A duration of diabetes prevalence longer than 10 years was associated with a high risk of OHCA compared to participants without diabetes. In terms of HbA1c level, there was a dose-dependent relationship between HbA1c level and OHCA risk. Our study emphasizes that DM diagnosis and its characteristics could affect the risk of OHCA, and delaying the onset of diabetes and appropriate glucose control of DM might reduce the risk of OHCA in patients with DM. Understanding the risks of DM on OHCA according to the characteristics of diabetes is essential to develop health policies and public interventions preventing cardiac arrests caused by diabetes.

Diabetes is a well-known risk factor for OHCA^5–9,17^, and the prevalence of DM was higher in OHCA cases than in controls in this study (31.3% in cases and 18.9% in controls, aOR (95% CI): 2.13 (1.64–2.75)). Several potential mechanisms were suggested to explain the association between DM and OHCA incidence. Diabetes is associated with coronary artery disease and related cardiovascular risks and is the most common cause of sudden cardiac arrest^18–20^. In addition, diabetes is associated with electrocardiographic abnormalities of electrical propagation in the myocardium, which could result in prolongation of the QT and QRS interval^17^. Diabetic autonomic neuropathy reportedly has a critical role in this mechanism^21^. An increased risk of hypoglycemic episodes has also been reported to be associated with arrhythmic events and sudden cardiac arrest^22^. Moreover, diabetes could result in the development of structural changes in the myocardium and interstitium in the diabetic heart, which is called diabetic cardiomyopathy and is associated with sudden cardiac arrest^23^.

In this study, we found that the various diagnostic characteristics (type of DM and prevalence duration) and treatment characteristics (treatment modality and HbA1c level) of DM could modify the magnitude of risks for OHCA. By type of DM, the proportion of type I DM was smaller than that of type II DM, but the risk magnitude of OHCA was greater in the type I DM group than in the type II DM group compared to the no diabetes group (aORs (95% CIs): 5.26 (1.72–16.08) for type I DM and 1.63 (1.18–2.27) for type II DM). Although few studies about differences in the risks of OHCA incidence between types of DM have been published^10^, a previous study reported that the incidence of sudden cardiac death was higher in type I DM than in type II DM in patients aged 1–49 years^6^. According to the duration of diabetes, patient groups with a diabetes prevalence longer than 10 years had a higher risk of OHCA compared to the no diabetes group. A previous case–control study also reported that the duration of diabetes prevalence was longer in sudden cardiac arrest patients with DM than in age-, gender-, and index year-matched controls with DM^17^.

Among the treatment modalities for DM, diabetic patients receiving insulin therapy showed the highest risk of OHCA (aOR (95% CI): 6.63 (3.04–14.44)), whereas diabetic patients receiving oral hypoglycemic agents showed the lowest risk (aOR (95% CI): 1.47 (1.08–2.01)) compared to patients without diabetes. The risk magnitudes on OHCA for patients with no treatment and lifestyle modification were smaller than those of diabetes patients with insulin therapy (aORs (95% CIs): 2.76 (1.01–7.58) and 2.62 (1.14–6.06)); however, there was no significant difference between the effect sizes. This result was not in concordance with the results from a previous phase I CAPTURES study in which the nonpharmacologic therapy group showed the highest risk^5^. DM with nonpharmacologic therapy might be heterogeneous because both the early phase after the diagnosis of diabetes and the advanced phase with poor compliance could belong to this group^24^. In terms of HbA1c level, we found that there was a dose-dependent relationship between HbA1c and the risk of OHCA, similar to previous studies^25,26^. Strict regulation of blood glucose would reduce the risk of OHCA in patients with DM^27^.

Various diagnostic and therapeutic characteristics of DM are interrelated^28^. For example, insulin therapy is more common in patients with type I DM, a long duration of DM prevalence, and high HbA1c levels. Patients with those factors had a greater risk for OHCA. For those high-risk groups, adequate glycemic control and strict risk management could reduce the risk of fatal cardiovascular complications, even in patients with type I DM^6^, a long duration of DM prevalence with microvascular disease^7^, and insulin therapy^29^. A recent study reported that preventive interventions for prolonged diabetes onset mitigate cardiometabolic risks, regardless of genetic risk^30^. Moreover, early cardiovascular risk monitoring and steady management have benefits for alleviation of disease burden among young patients who develop DM in the early period of their life^6^. Public interventions for delaying the onset of diabetes among at-risk populations and monitoring programs for adequate glucose control in patients diagnosed with DM from the early phase immediately after diagnosis will be key strategies to reduce the complications of DM and its risk for cardiac arrest.

This study has several limitations. First, information on the characteristics of diabetes, including diagnosis, type, duration of diabetes, and treatment modality, was collected from family members in most OHCA cases because most OHCA patients were not alert and direct history taking was not feasible. However, this information was obtained directly from subjects in the community-based control group. This difference might lead to information bias and under- or overestimation of diabetes prevalence and its severity among the OHCA case group. Second, the presence of microvascular or macrovascular complications, diabetic autonomic neuropathy, and electrocardiographic abnormalities were not evaluated. These factors may have yielded different effect sizes. Third, because the consent of subjects or family members was necessary for enrollment, OHCA patients who achieved successful resuscitation or were admitted to the hospital were more likely to be enrolled than patients who died in the ED. Fourth, the CAPTURES project enrolled OHCA patients with presumed cardiac etiology. Caution should be taken when interpreting this study results given the significant limitations. Last, the study design was a case–control study, not an intervention trial. There may be significant potential biases that were not controlled for. In addition, there was a possibility of misclassification for matching variables.

## Conclusions

In this multicenter prospective case–control study, we found that diabetes was associated with an increased risk of OHCA. The risk for the cardiac arrest varied according to the diagnostic and therapeutic characteristics of diabetes, and a long prevalence duration and high HbA1c level were strongly associated with the OHCA risk compared to patients without diabetes. Our study emphasizes that public interventions for delaying the onset of DM in at-risk populations and adequate glucose control from the early phase for diabetic patients would be key strategies to reduce the risks of fatal cardiovascular complications, including OHCA.

## Supplementary Information

Supplementary Information.

## Data Availability

Data were obtained from the Korea Disease Control and Prevention Agency.
