# Supplementary material for: Diagnostic and therapeutic characteristics of diabetes mellitus and risk of out-of-hospital cardiac arrest
Source: Sci Rep. 2022 Jan 25;12:1293. doi: 10.1038/s41598-022-05390-w (PMC8789864; doi:10.1038/s41598-022-05390-w)
Supplement: Supplementary file 1 — Supplementary Information. [file 41598_2022_5390_MOESM1_ESM.pdf]

Supplementary Table 1. Characteristics of study population by type of diabetes mellitus

	Total	No DM	Diabetes mellitus		
			Type I	Type II	Unknown
	N (%)	N (%)	N (%)		
Total	2316	1782	32	358	144
OHCA case	772 (33.3)	530 (29.7)	26 (81.3)	152 (42.5)	64 (44.4)
Control	1544 (66.7)	1252 (70.3)	6 (18.8)	206 (57.5)	80 (55.6)
Sex, male	1671 (72.2)	1290 (72.4)	27 (84.4)	252 (70.4)	102 (70.8)
Age, mean (SD), year	58.2 (12.0)	56.5 (12.4)	63.2 (8.6)	63.0 (8.2)	66.9 (8.4)
Age, year					
19–29	45 (1.9)	45 (2.5)	0 (0.0)	0 (0.0)	0 (0.0)
30–39	132 (5.7)	132 (7.4)	0 (0.0)	0 (0.0)	0 (0.0)
40–49	372 (16.1)	343 (19.2)	4 (12.5)	18 (5.0)	7 (4.9)
50–59	573 (24.7)	458 (25.7)	4 (12.5)	91 (25.4)	20 (13.9)
60–69	777 (33.5)	522 (29.3)	17 (53.1)	173 (48.3)	65 (45.1)
70–79	417 (18.0)	282 (15.8)	7 (21.9)	76 (21.2)	52 (36.1)
Urbanization level of residence					
Metropolitan	1152 (49.7)	873 (49.0)	22 (68.8)	154 (43.0)	103 (71.5)
Health behavior					
Current smoker	588 (25.4)	460 (25.8)	12 (37.5)	80 (22.3)	36 (25.0)
Obesity	217 (9.4)	151 (8.5)	1 (3.1)	37 (10.3)	28 (19.4)
Regular exercise during the last year	1195 (51.6)	946 (53.1)	15 (46.9)	155 (43.3)	79 (54.9)
Comorbidity					
Hypertension	911 (39.3)	560 (31.4)	15 (46.9)	231 (64.5)	105 (72.9)
Myocardial infarction	80 (3.5)	37 (2.1)	7 (21.9)	27 (7.5)	9 (6.3)
Stroke	102 (4.4)	64 (3.6)	9 (28.1)	22 (6.1)	7 (4.9)
Dyslipidemia	504 (21.8)	330 (18.5)	8 (25.0)	107 (29.9)	59 (41.0)
Arrhythmia	105 (4.5)	72 (4.0)	4 (12.5)	16 (4.5)	13 (9.0)
Diabetes mellitus					
Duration of diabetes, years					
0–3	114 (21.3)	-	5 (15.6)	90 (25.1)	19 (13.2)
4–10	130 (24.3)	-	7 (21.9)	98 (27.4)	25 (17.4)
11–18	77 (14.4)	-	3 (9.4)	54 (15.1)	20 (13.9)
19–	85 (15.9)	-	6 (18.8)	57 (15.9)	22 (15.3)
Unknown	128 (24.0)	-	11 (34.4)	59 (16.5)	58 (40.3)
Median (IQR)	10 (3-17)	-	10 (4-19)	10 (3-15)	10 (4-19)
Treatment of diabetes					
No treatment	28 (5.2)	-	1 (3.1)	16 (4.5)	11 (7.6)
Lifestyle modification	30 (5.6)	-	0 (0.0)	19 (5.3)	11 (7.6)
Oral hypoglycemic agent	372 (69.7)	-	20 (62.5)	266 (74.3)	86 (59.7)
Insulin	56 (10.5)	-	5 (15.6)	35 (9.8)	16 (11.1)
Unknown	48 (9.0)	-	6 (18.8)	22 (6.1)	20 (13.9)
HbA1c, %			-		
0–5.6	37 (6.9)	-	0 (0.0)	28 (7.8)	9 (6.3)
5.7–6.4	109 (20.4)	-	4 (12.5)	68 (19.0)	37 (25.7)
6.5–	270 (50.6)	-	17 (53.1)	181 (50.6)	72 (50.0)
Unknown	118 (22.1)	-	11 (34.4)	81 (22.6)	26 (18.1)
Median (IQR)	6.7 (6.2–7.6)	-	6.9 (6.6–7.3)	6.8 (6.2–7.5)	6.6 (6.1–7.9)

DM, Diabetes mellitus; IQR, interquartile range
